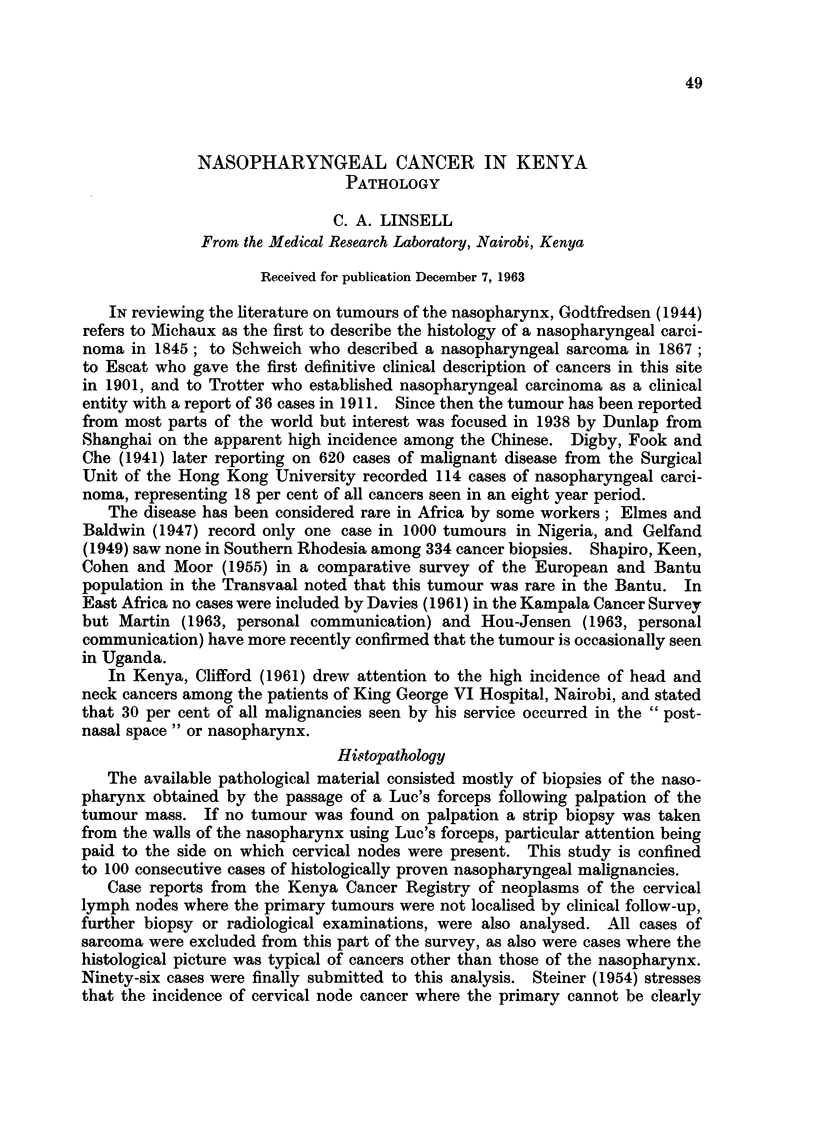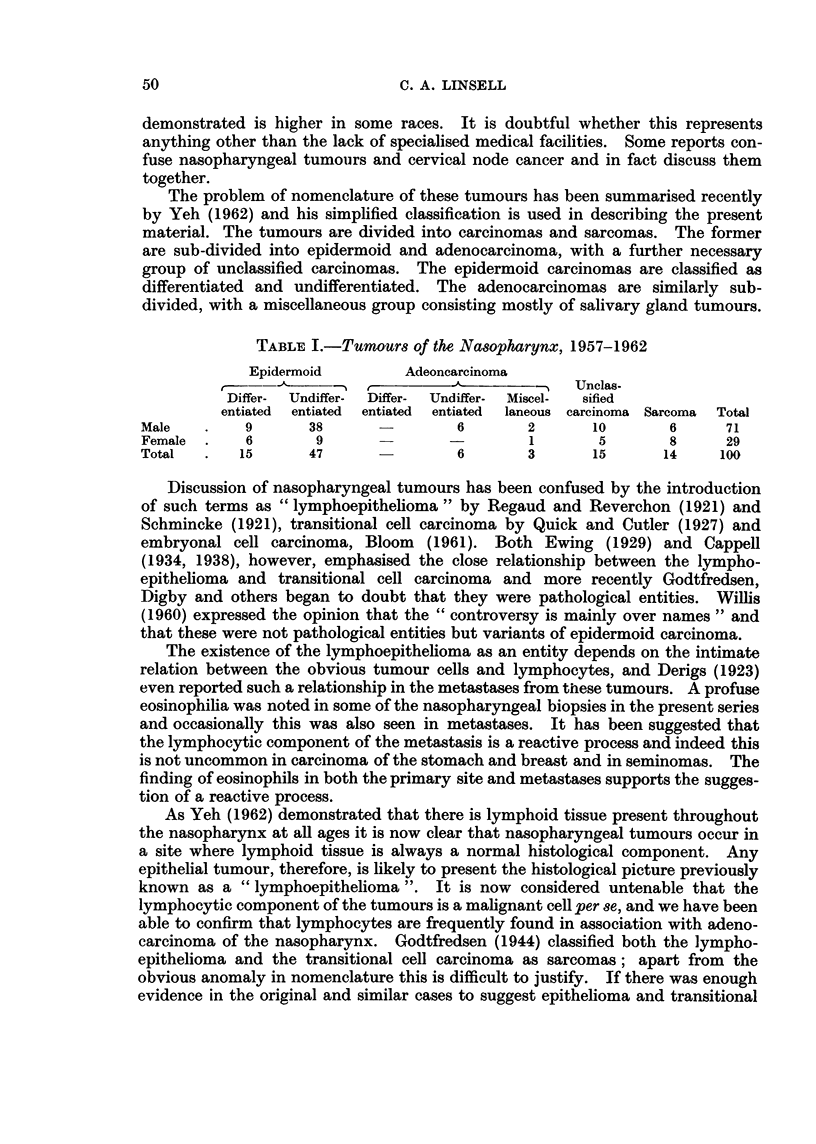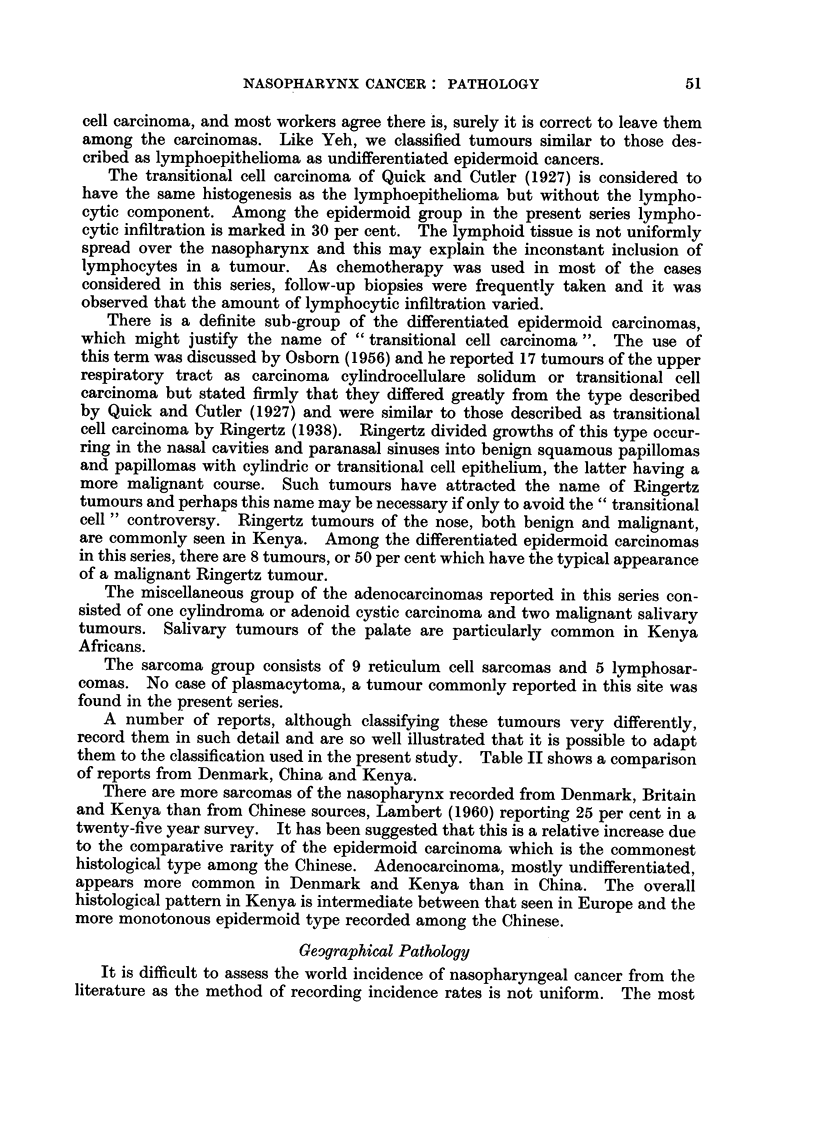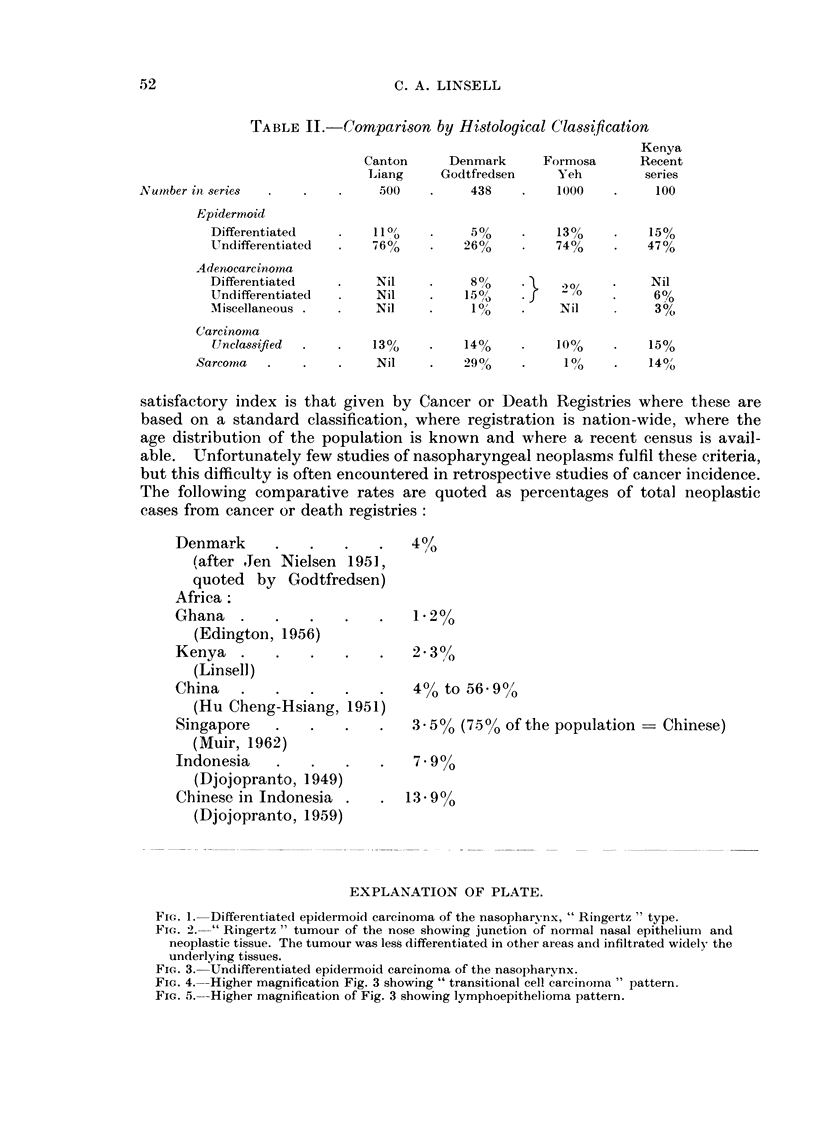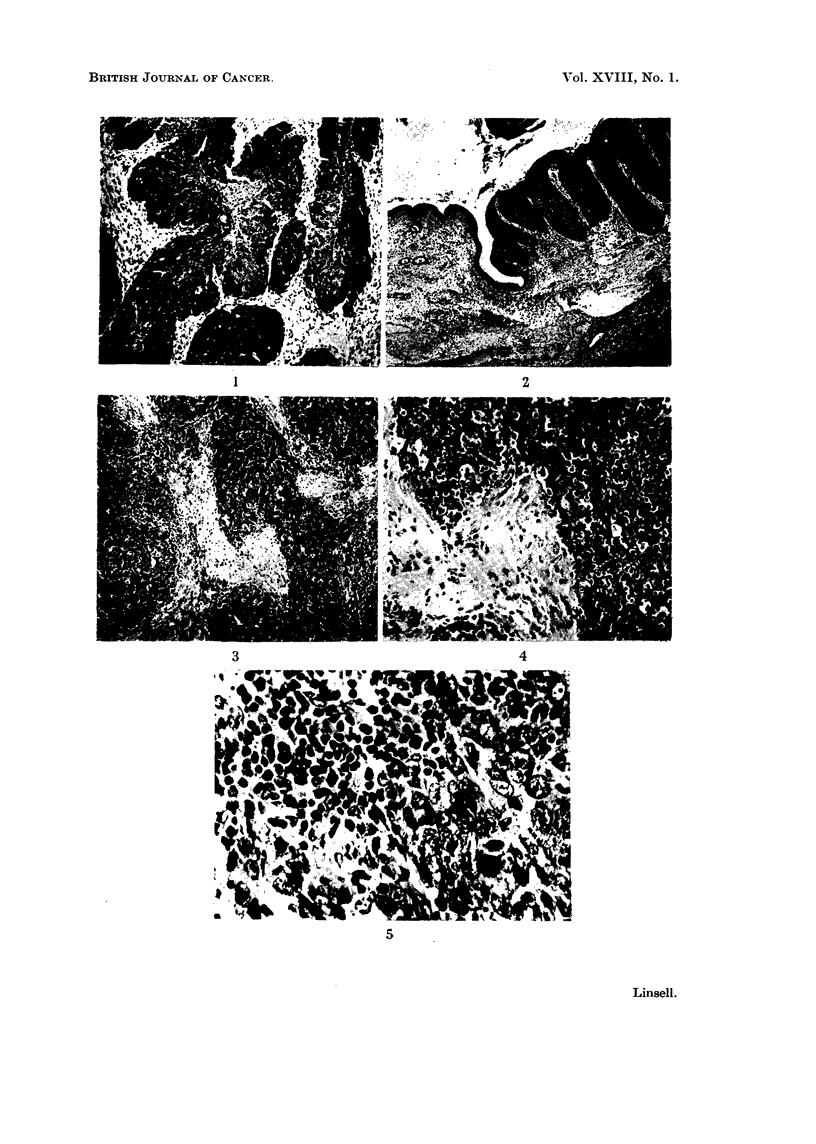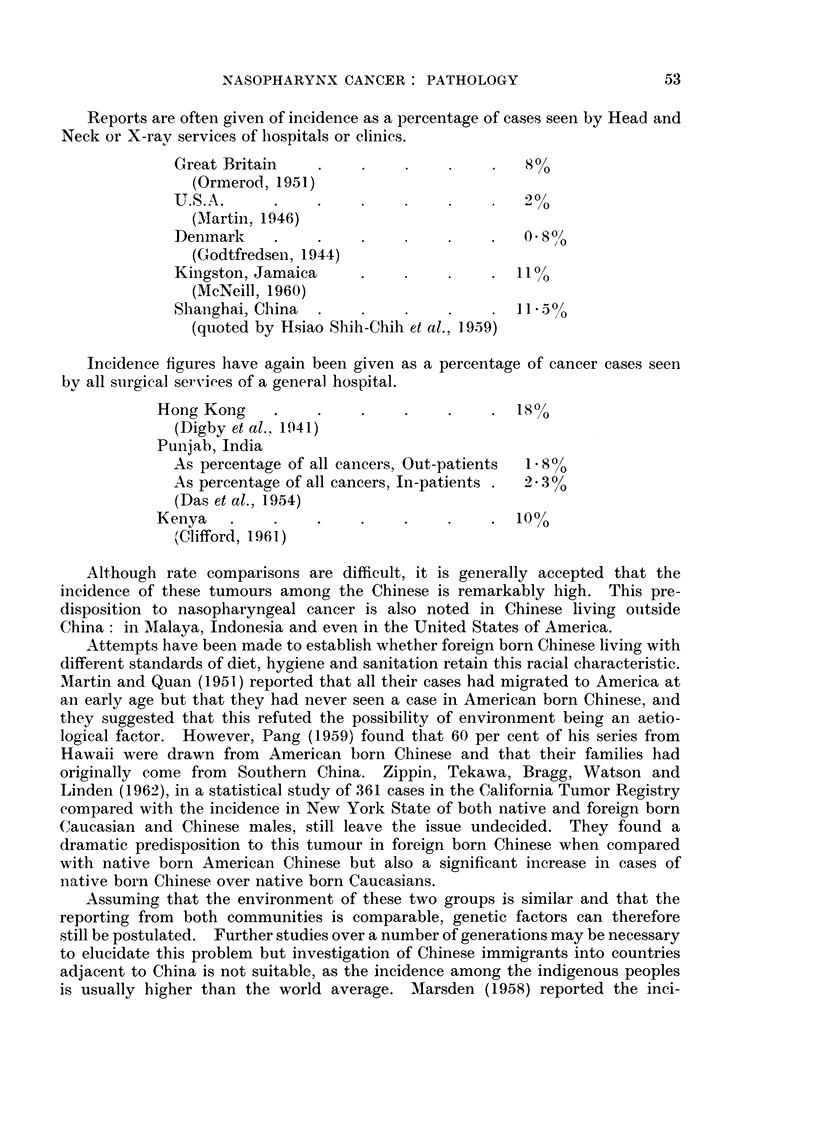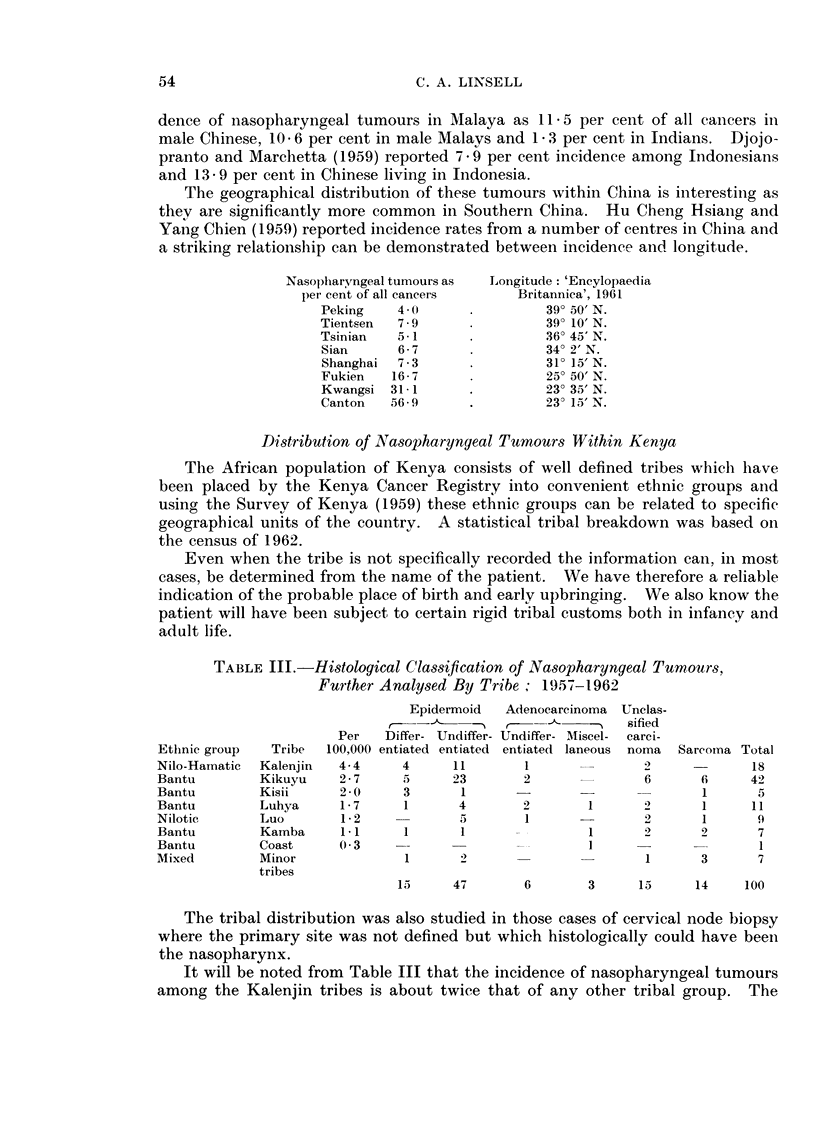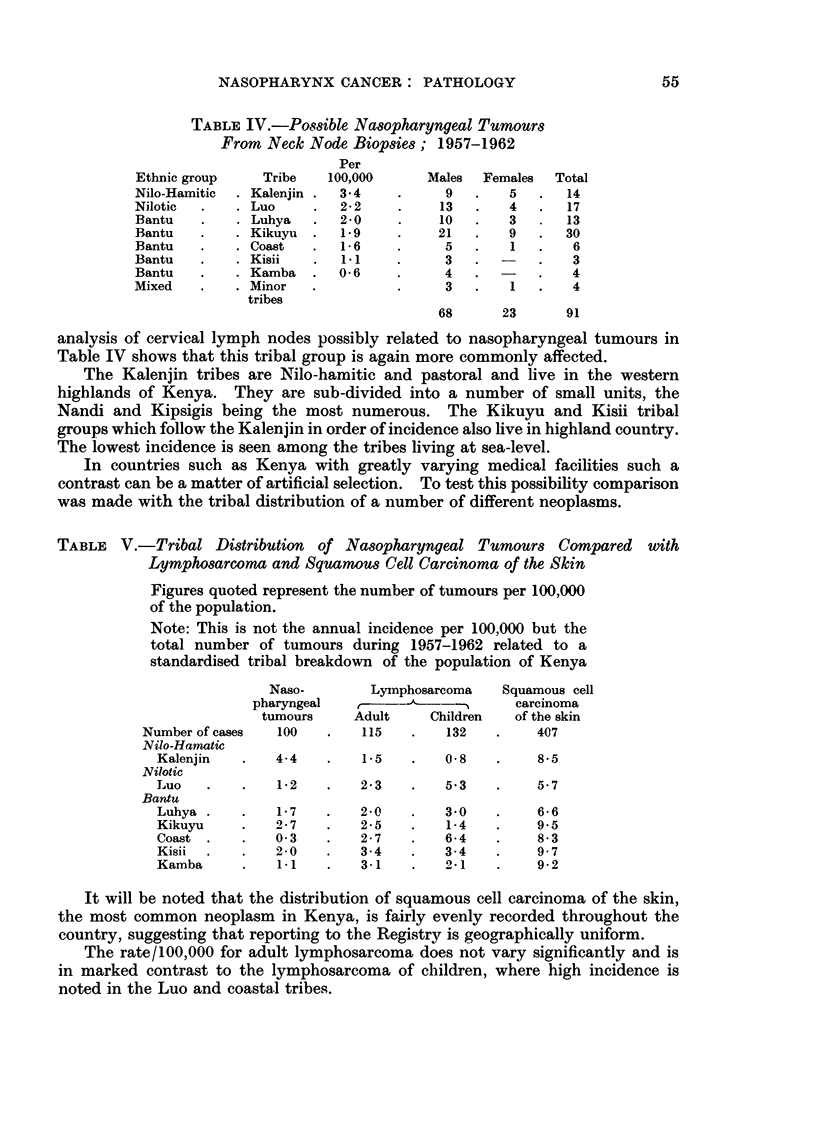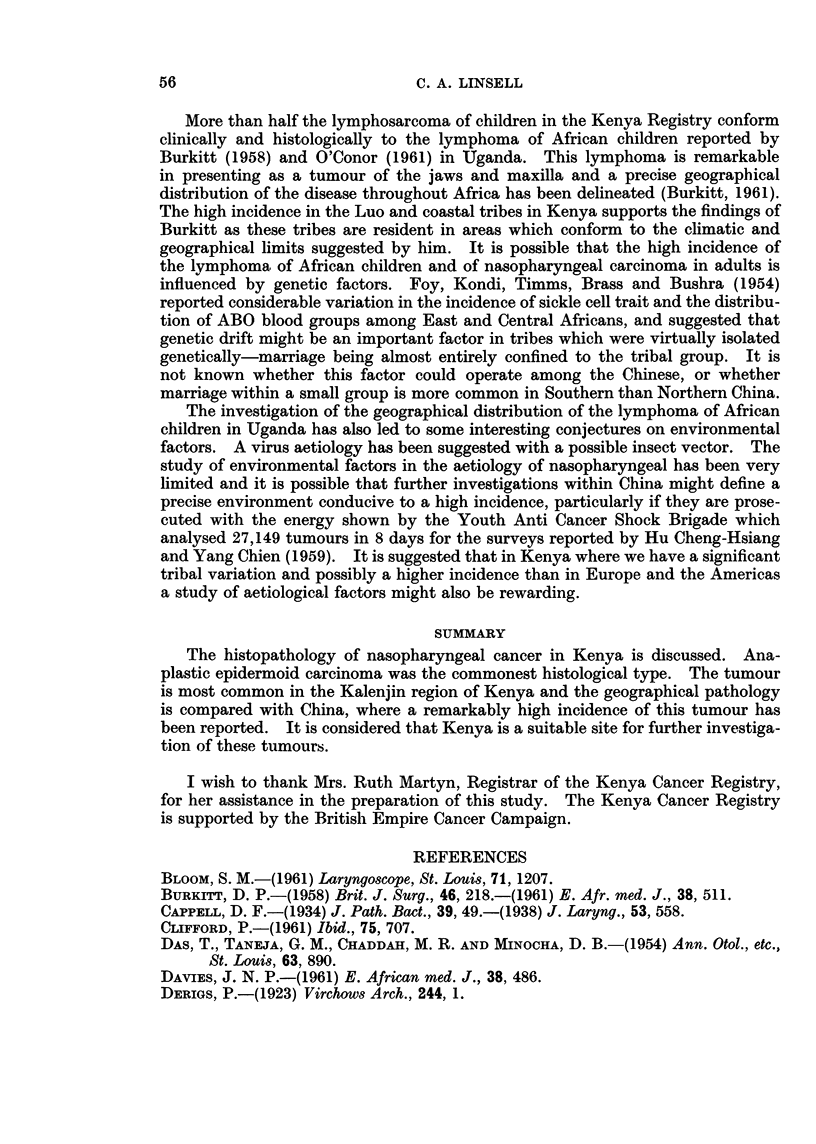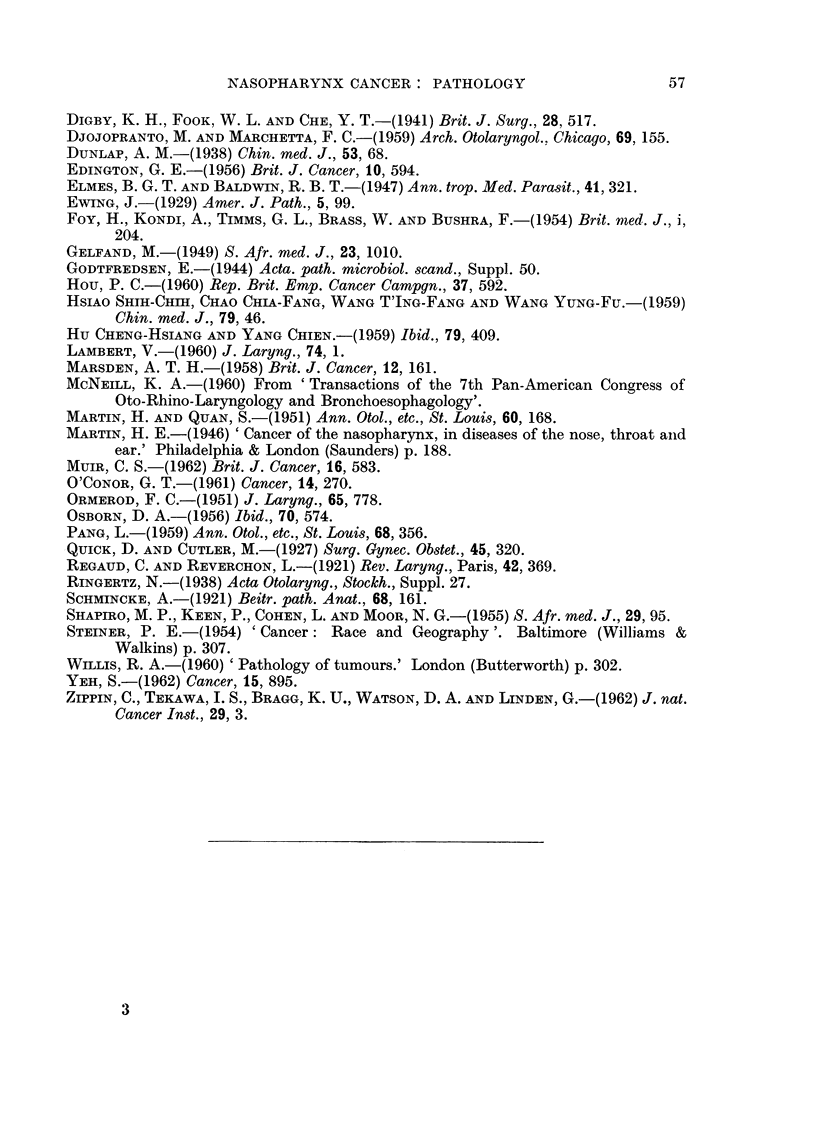# Nasopharyngeal Cancer in Kenya

**DOI:** 10.1038/bjc.1964.5

**Published:** 1964-03

**Authors:** C. A. Linsell

## Abstract

**Images:**


### NASOPHARYNGEAL CANCER IN KENYA
### Pathology

C. A. LINSELL

*From the Medical Research Laboratory, Nairobi, Kenya*

Received for publication December 7, 1963

In reviewing the literature on tumours of the nasopharynx, Godtfredsen (1944) refers to Michaux as the first to describe the histology of a nasopharyngeal carcinoma in 1845 ; to Schweich who described a nasopharyngeal sarcoma in 1867 ; to Escat who gave the first definitive clinical description of cancers in this site in 1901, and to Trotter who established nasopharyngeal carcinoma as a clinical entity with a report of 36 cases in 1911. Since then the tumour has been reported from most parts of the world but interest was focused in 1938 by Dunlap from Shanghai on the apparent high incidence among the Chinese. Digby, Fook and Che (1941) later reporting on 620 cases of malignant disease from the Surgical Unit of the Hong Kong University recorded 114 cases of nasopharyngeal carcinoma, representing 18 per cent of all cancers seen in an eight year period.

The disease has been considered rare in Africa by some workers ; Elmes and Baldwin (1947) record only one case in 1000 tumours in Nigeria, and Gelfand (1949) saw none in Southern Rhodesia among 334 cancer biopsies. Shapiro, Keen, Cohen and Moor (1955) in a comparative survey of the European and Bantu population in the Transvaal noted that this tumour was rare in the Bantu. In East Africa no cases were included by Davies (1961) in the Kampala Cancer Survey but Martin (1963, personal communication) and Hou-Jensen (1963, personal communication) have more recently confirmed that the tumour is occasionally seen in Uganda.

In Kenya, Clifford (1961) drew attention to the high incidence of head and neck cancers among the patients of King George VI Hospital, Nairobi, and stated that 30 per cent of all malignancies seen by his service occurred in the " post-nasal space " or nasopharynx.

### *Histopathology*

The available pathological material consisted mostly of biopsies of the nasopharynx obtained by the passage of a Luc's forceps following palpation of the tumour mass. If no tumour was found on palpation a strip biopsy was taken from the walls of the nasopharynx using Luc's forceps, particular attention being paid to the side on which cervical nodes were present. This study is confined to 100 consecutive cases of histologically proven nasopharyngeal malignancies.

Case reports from the Kenya Cancer Registry of neoplasms of the cervical lymph nodes where the primary tumours were not localised by clinical follow-up, further biopsy or radiological examinations, were also analysed. All cases of sarcoma were excluded from this part of the survey, as also were cases where the histological picture was typical of cancers other than those of the nasopharynx. Ninety-six cases were finally submitted to this analysis. Steiner (1954) stresses that the incidence of cervical node cancer where the primary cannot be clearly demonstrated is higher in some races. It is doubtful whether this represents anything other than the lack of specialised medical facilities. Some reports confuse nasopharyngeal tumours and cervical node cancer and in fact discuss them together.

The problem of nomenclature of these tumours has been summarised recently by Yeh (1962) and his simplified classification is used in describing the present material. The tumours are divided into carcinomas and sarcomas. The former are sub-divided into epidermoid and adenocarcinoma, with a further necessary group of unclassified carcinomas. The epidermoid carcinomas are classified as differentiated and undifferentiated. The adenocarcinomas are similarly subdivided, with a miscellaneous group consisting mostly of salivary gland tumours.

TABLE I.—*Tumours of the Nasopharynx*, 1957–1962

| | Epidermoid | | Adeoncarcinoma | | | | | |
|---|---|---|---|---|---|---|---|---|
| | Differentiated | Undifferentiated | Differentiated | Undifferentiated | Miscellaneous | Unclassified carcinoma | Sarcoma | Total |
| Male | 9 | 38 | — | 6 | 2 | 10 | 6 | 71 |
| Female | 6 | 9 | — | — | 1 | 5 | 8 | 29 |
| Total | 15 | 47 | — | 6 | 3 | 15 | 14 | 100 |

Discussion of nasopharyngeal tumours has been confused by the introduction of such terms as " lymphoepithelioma " by Regaud and Reverchon (1921) and Schmincke (1921), transitional cell carcinoma by Quick and Cutler (1927) and embryonal cell carcinoma, Bloom (1961). Both Ewing (1929) and Cappell (1934, 1938), however, emphasised the close relationship between the lymphoepithelioma and transitional cell carcinoma and more recently Godtfredsen, Digby and others began to doubt that they were pathological entities. Willis (1960) expressed the opinion that the " controversy is mainly over names " and that these were not pathological entities but variants of epidermoid carcinoma.

The existence of the lymphoepithelioma as an entity depends on the intimate relation between the obvious tumour cells and lymphocytes, and Derigs (1923) even reported such a relationship in the metastases from these tumours. A profuse eosinophilia was noted in some of the nasopharyngeal biopsies in the present series and occasionally this was also seen in metastases. It has been suggested that the lymphocytic component of the metastasis is a reactive process and indeed this is not uncommon in carcinoma of the stomach and breast and in seminomas. The finding of eosinophils in both the primary site and metastases supports the suggestion of a reactive process.

As Yeh (1962) demonstrated that there is lymphoid tissue present throughout the nasopharynx at all ages it is now clear that nasopharyngeal tumours occur in a site where lymphoid tissue is always a normal histological component. Any epithelial tumour, therefore, is likely to present the histological picture previously known as a " lymphoepithelioma ". It is now considered untenable that the lymphocytic component of the tumours is a malignant cell *per se*, and we have been able to confirm that lymphocytes are frequently found in association with adenocarcinoma of the nasopharynx. Godtfredsen (1944) classified both the lymphoepithelioma and the transitional cell carcinoma as sarcomas ; apart from the obvious anomaly in nomenclature this is difficult to justify. If there was enough evidence in the original and similar cases to suggest epithelioma and transitional cell carcinoma, and most workers agree there is, surely it is correct to leave them among the carcinomas. Like Yeh, we classified tumours similar to those described as lymphoepithelioma as undifferentiated epidermoid cancers.

The transitional cell carcinoma of Quick and Cutler (1927) is considered to have the same histogenesis as the lymphoepithelioma but without the lymphocytic component. Among the epidermoid group in the present series lymphocytic infiltration is marked in 30 per cent. The lymphoid tissue is not uniformly spread over the nasopharynx and this may explain the inconstant inclusion of lymphocytes in a tumour. As chemotherapy was used in most of the cases considered in this series, follow-up biopsies were frequently taken and it was observed that the amount of lymphocytic infiltration varied.

There is a definite sub-group of the differentiated epidermoid carcinomas, which might justify the name of " transitional cell carcinoma ". The use of this term was discussed by Osborn (1956) and he reported 17 tumours of the upper respiratory tract as carcinoma cylindrocellulare solidum or transitional cell carcinoma but stated firmly that they differed greatly from the type described by Quick and Cutler (1927) and were similar to those described as transitional cell carcinoma by Ringertz (1938). Ringertz divided growths of this type occurring in the nasal cavities and paranasal sinuses into benign squamous papillomas and papillomas with cylindric or transitional cell epithelium, the latter having a more malignant course. Such tumours have attracted the name of Ringertz tumours and perhaps this name may be necessary if only to avoid the " transitional cell " controversy. Ringertz tumours of the nose, both benign and malignant, are commonly seen in Kenya. Among the differentiated epidermoid carcinomas in this series, there are 8 tumours, or 50 per cent which have the typical appearance of a malignant Ringertz tumour.

The miscellaneous group of the adenocarcinomas reported in this series consisted of one cylindroma or adenoid cystic carcinoma and two malignant salivary tumours. Salivary tumours of the palate are particularly common in Kenya Africans.

The sarcoma group consists of 9 reticulum cell sarcomas and 5 lymphosarcomas. No case of plasmacytoma, a tumour commonly reported in this site was found in the present series.

A number of reports, although classifying these tumours very differently, record them in such detail and are so well illustrated that it is possible to adapt them to the classification used in the present study. Table II shows a comparison of reports from Denmark, China and Kenya.

There are more sarcomas of the nasopharynx recorded from Denmark, Britain and Kenya than from Chinese sources, Lambert (1960) reporting 25 per cent in a twenty-five year survey. It has been suggested that this is a relative increase due to the comparative rarity of the epidermoid carcinoma which is the commonest histological type among the Chinese. Adenocarcinoma, mostly undifferentiated, appears more common in Denmark and Kenya than in China. The overall histological pattern in Kenya is intermediate between that seen in Europe and the more monotonous epidermoid type recorded among the Chinese.

### *Geographical Pathology*

It is difficult to assess the world incidence of nasopharyngeal cancer from the literature as the method of recording incidence rates is not uniform. The most

TABLE II.—*Comparison by Histological Classification*

| | Canton Liang | Denmark Godtfredsen | Formosa Yeh | Kenya Recent series |
|---|---|---|---|---|
| *Number in series* | 500 | 438 | 1000 | 100 |
| *Epidermoid* | | | | |
| Differentiated | 11% | 5% | 13% | 15% |
| Undifferentiated | 76% | 26% | 74% | 47% |
| *Adenocarcinoma* | | | | |
| Differentiated | Nil | 8% | } 2% | Nil |
| Undifferentiated | Nil | 15% | | 6% |
| Miscellaneous | Nil | 1% | Nil | 3% |
| *Carcinoma Unclassified* | 13% | 14% | 10% | 15% |
| *Sarcoma* | Nil | 29% | 1% | 14% |

satisfactory index is that given by Cancer or Death Registries where these are based on a standard classification, where registration is nation-wide, where the age distribution of the population is known and where a recent census is available. Unfortunately few studies of nasopharyngeal neoplasms fulfil these criteria, but this difficulty is often encountered in retrospective studies of cancer incidence. The following comparative rates are quoted as percentages of total neoplastic cases from cancer or death registries :

| | |
|---|---|
| Denmark (after Jen Nielsen 1951, quoted by Godtfredsen) | 4% |
| Africa : | |
| Ghana (Edington, 1956) | 1·2% |
| Kenya (Linsell) | 2·3% |
| China (Hu Cheng-Hsiang, 1951) | 4% to 56·9% |
| Singapore (Muir, 1962) | 3·5% (75% of the population = Chinese) |
| Indonesia (Djojopranto, 1949) | 7·9% |
| Chinese in Indonesia (Djojopranto, 1959) | 13·9% |

EXPLANATION OF PLATE.

FIG. 1.—Differentiated epidermoid carcinoma of the nasopharynx, " Ringertz " type.
FIG. 2.—" Ringertz " tumour of the nose showing junction of normal nasal epithelium and neoplastic tissue. The tumour was less differentiated in other areas and infiltrated widely the underlying tissues.
FIG. 3.—Undifferentiated epidermoid carcinoma of the nasopharynx.
FIG. 4.—Higher magnification Fig. 3 showing " transitional cell carcinoma " pattern.
FIG. 5.—Higher magnification of Fig. 3 showing lymphoepithelioma pattern.

1

2

3

4

5

Reports are often given of incidence as a percentage of cases seen by Head and Neck or X-ray services of hospitals or clinics.

| | |
|---|---|
| Great Britain (Ormerod, 1951) | 8% |
| U.S.A. (Martin, 1946) | 2% |
| Denmark (Godtfredsen, 1944) | 0·8% |
| Kingston, Jamaica (McNeill, 1960) | 11% |
| Shanghai, China (quoted by Hsiao Shih-Chih *et al.*, 1959) | 11·5% |

Incidence figures have again been given as a percentage of cancer cases seen by all surgical services of a general hospital.

| | |
|---|---|
| Hong Kong (Digby *et al.*, 1941) | 18% |
| Punjab, India | |
| As percentage of all cancers, Out-patients | 1·8% |
| As percentage of all cancers, In-patients | 2·3% |
| (Das *et al.*, 1954) | |
| Kenya (Clifford, 1961) | 10% |

Although rate comparisons are difficult, it is generally accepted that the incidence of these tumours among the Chinese is remarkably high. This predisposition to nasopharyngeal cancer is also noted in Chinese living outside China : in Malaya, Indonesia and even in the United States of America.

Attempts have been made to establish whether foreign born Chinese living with different standards of diet, hygiene and sanitation retain this racial characteristic. Martin and Quan (1951) reported that all their cases had migrated to America at an early age but that they had never seen a case in American born Chinese, and they suggested that this refuted the possibility of environment being an aetiological factor. However, Pang (1959) found that 60 per cent of his series from Hawaii were drawn from American born Chinese and that their families had originally come from Southern China. Zippin, Tekawa, Bragg, Watson and Linden (1962), in a statistical study of 361 cases in the California Tumor Registry compared with the incidence in New York State of both native and foreign born Caucasian and Chinese males, still leave the issue undecided. They found a dramatic predisposition to this tumour in foreign born Chinese when compared with native born American Chinese but also a significant increase in cases of native born Chinese over native born Caucasians.

Assuming that the environment of these two groups is similar and that the reporting from both communities is comparable, genetic factors can therefore still be postulated. Further studies over a number of generations may be necessary to elucidate this problem but investigation of Chinese immigrants into countries adjacent to China is not suitable, as the incidence among the indigenous peoples is usually higher than the world average. Marsden (1958) reported the incidence of nasopharyngeal tumours in Malaya as 11·5 per cent of all cancers in male Chinese, 10·6 per cent in male Malays and 1·3 per cent in Indians. Djojopranto and Marchetta (1959) reported 7·9 per cent incidence among Indonesians and 13·9 per cent in Chinese living in Indonesia.

The geographical distribution of these tumours within China is interesting as they are significantly more common in Southern China. Hu Cheng Hsiang and Yang Chien (1959) reported incidence rates from a number of centres in China and a striking relationship can be demonstrated between incidence and longitude.

| Nasopharyngeal tumours as per cent of all cancers | | | Longitude : 'Encylopaedia Britannica', 1961 |
|---|---|---|---|
| Peking | 4·0 | . | 39° 50′ N. |
| Tientsen | 7·9 | . | 39° 10′ N. |
| Tsinian | 5·1 | . | 36° 45′ N. |
| Sian | 6·7 | . | 34° 2′ N. |
| Shanghai | 7·3 | . | 31° 15′ N. |
| Fukien | 16·7 | . | 25° 50′ N. |
| Kwangsi | 31·1 | . | 23° 35′ N. |
| Canton | 56·9 | . | 23° 15′ N. |

### *Distribution of Nasopharyngeal Tumours Within Kenya*

The African population of Kenya consists of well defined tribes which have been placed by the Kenya Cancer Registry into convenient ethnic groups and using the Survey of Kenya (1959) these ethnic groups can be related to specific geographical units of the country. A statistical tribal breakdown was based on the census of 1962.

Even when the tribe is not specifically recorded the information can, in most cases, be determined from the name of the patient. We have therefore a reliable indication of the probable place of birth and early upbringing. We also know the patient will have been subject to certain rigid tribal customs both in infancy and adult life.

TABLE III.—*Histological Classification of Nasopharyngeal Tumours, Further Analysed By Tribe ;* 1957–1962

| | | | Epidermoid | | Adenocarcinoma | | | | |
|---|---|---|---|---|---|---|---|---|---|
| Ethnic group | Tribe | Per 100,000 | Differentiated | Undifferentiated | Undifferentiated | Miscellaneous | Unclassified carcinoma | Sarcoma | Total |
| Nilo-Hamatic | Kalenjin | 4·4 | 4 | 11 | 1 | — | 2 | — | 18 |
| Bantu | Kikuyu | 2·7 | 5 | 23 | 2 | — | 6 | 6 | 42 |
| Bantu | Kisii | 2·0 | 3 | 1 | — | — | — | 1 | 5 |
| Bantu | Luhya | 1·7 | 1 | 4 | 2 | 1 | 2 | 1 | 11 |
| Nilotic | Luo | 1·2 | — | 5 | 1 | — | 2 | 1 | 9 |
| Bantu | Kamba | 1·1 | 1 | 1 | — | 1 | 2 | 2 | 7 |
| Bantu | Coast | 0·3 | — | — | — | 1 | — | — | 1 |
| Mixed | Minor tribes | | 1 | 2 | — | — | 1 | 3 | 7 |
| | | | 15 | 47 | 6 | 3 | 15 | 14 | 100 |

The tribal distribution was also studied in those cases of cervical node biopsy where the primary site was not defined but which histologically could have been the nasopharynx.

It will be noted from Table III that the incidence of nasopharyngeal tumours among the Kalenjin tribes is about twice that of any other tribal group. The

TABLE IV.—*Possible Nasopharyngeal Tumours From Neck Node Biopsies ;* 1957–1962

| Ethnic group | Tribe | Per 100,000 | Males | Females | Total |
|---|---|---|---|---|---|
| Nilo-Hamitic | Kalenjin | 3·4 | 9 | 5 | 14 |
| Nilotic | Luo | 2·2 | 13 | 4 | 17 |
| Bantu | Luhya | 2·0 | 10 | 3 | 13 |
| Bantu | Kikuyu | 1·9 | 21 | 9 | 30 |
| Bantu | Coast | 1·6 | 5 | 1 | 6 |
| Bantu | Kisii | 1·1 | 3 | — | 3 |
| Bantu | Kamba | 0·6 | 4 | — | 4 |
| Mixed | Minor tribes | | 3 | 1 | 4 |
| | | | 68 | 23 | 91 |

analysis of cervical lymph nodes possibly related to nasopharyngeal tumours in Table IV shows that this tribal group is again more commonly affected.

The Kalenjin tribes are Nilo-hamitic and pastoral and live in the western highlands of Kenya. They are sub-divided into a number of small units, the Nandi and Kipsigis being the most numerous. The Kikuyu and Kisii tribal groups which follow the Kalenjin in order of incidence also live in highland country. The lowest incidence is seen among the tribes living at sea-level.

In countries such as Kenya with greatly varying medical facilities such a contrast can be a matter of artificial selection. To test this possibility comparison was made with the tribal distribution of a number of different neoplasms.

TABLE V.—*Tribal Distribution of Nasopharyngeal Tumours Compared with Lymphosarcoma and Squamous Cell Carcinoma of the Skin*

Figures quoted represent the number of tumours per 100,000 of the population.

Note: This is not the annual incidence per 100,000 but the total number of tumours during 1957–1962 related to a standardised tribal breakdown of the population of Kenya

| | Naso-pharyngeal tumours | Lymphosarcoma | | Squamous cell carcinoma of the skin |
|---|---|---|---|---|
| | | Adult | Children | |
| Number of cases | 100 | 115 | 132 | 407 |
| *Nilo-Hamatic* | | | | |
| Kalenjin | 4·4 | 1·5 | 0·8 | 8·5 |
| *Nilotic* | | | | |
| Luo | 1·2 | 2·3 | 5·3 | 5·7 |
| *Bantu* | | | | |
| Luhya | 1·7 | 2·0 | 3·0 | 6·6 |
| Kikuyu | 2·7 | 2·5 | 1·4 | 9·5 |
| Coast | 0·3 | 2·7 | 6·4 | 8·3 |
| Kisii | 2·0 | 3·4 | 3·4 | 9·7 |
| Kamba | 1·1 | 3·1 | 2·1 | 9·2 |

It will be noted that the distribution of squamous cell carcinoma of the skin, the most common neoplasm in Kenya, is fairly evenly recorded throughout the country, suggesting that reporting to the Registry is geographically uniform.

The rate/100,000 for adult lymphosarcoma does not vary significantly and is in marked contrast to the lymphosarcoma of children, where high incidence is noted in the Luo and coastal tribes.

More than half the lymphosarcoma of children in the Kenya Registry conform clinically and histologically to the lymphoma of African children reported by Burkitt (1958) and O'Conor (1961) in Uganda. This lymphoma is remarkable in presenting as a tumour of the jaws and maxilla and a precise geographical distribution of the disease throughout Africa has been delineated (Burkitt, 1961). The high incidence in the Luo and coastal tribes in Kenya supports the findings of Burkitt as these tribes are resident in areas which conform to the climatic and geographical limits suggested by him. It is possible that the high incidence of the lymphoma of African children and of nasopharyngeal carcinoma in adults is influenced by genetic factors. Foy, Kondi, Timms, Brass and Bushra (1954) reported considerable variation in the incidence of sickle cell trait and the distribution of ABO blood groups among East and Central Africans, and suggested that genetic drift might be an important factor in tribes which were virtually isolated genetically—marriage being almost entirely confined to the tribal group. It is not known whether this factor could operate among the Chinese, or whether marriage within a small group is more common in Southern than Northern China.

The investigation of the geographical distribution of the lymphoma of African children in Uganda has also led to some interesting conjectures on environmental factors. A virus aetiology has been suggested with a possible insect vector. The study of environmental factors in the aetiology of nasopharyngeal has been very limited and it is possible that further investigations within China might define a precise environment conducive to a high incidence, particularly if they are prosecuted with the energy shown by the Youth Anti Cancer Shock Brigade which analysed 27,149 tumours in 8 days for the surveys reported by Hu Cheng-Hsiang and Yang Chien (1959). It is suggested that in Kenya where we have a significant tribal variation and possibly a higher incidence than in Europe and the Americas a study of aetiological factors might also be rewarding.

SUMMARY

The histopathology of nasopharyngeal cancer in Kenya is discussed. Anaplastic epidermoid carcinoma was the commonest histological type. The tumour is most common in the Kalenjin region of Kenya and the geographical pathology is compared with China, where a remarkably high incidence of this tumour has been reported. It is considered that Kenya is a suitable site for further investigation of these tumours.

I wish to thank Mrs. Ruth Martyn, Registrar of the Kenya Cancer Registry, for her assistance in the preparation of this study. The Kenya Cancer Registry is supported by the British Empire Cancer Campaign.

### REFERENCES

Bloom, S. M.—(1961) *Laryngoscope, St. Louis*, **71**, 1207.
Burkitt, D. P.—(1958) *Brit. J. Surg.*, **46**, 218.—(1961) *E. Afr. med. J.*, **38**, 511.
Cappell, D. F.—(1934) *J. Path. Bact.*, **39**, 49.—(1938) *J. Laryng.*, **53**, 558.
Clifford, P.—(1961) *Ibid.*, **75**, 707.
Das, T., Taneja, G. M., Chaddah, M. R. and Minocha, D. B.—(1954) *Ann. Otol., etc., St. Louis*, **63**, 890.
Davies, J. N. P.—(1961) *E. African med. J.*, **38**, 486.
Derigs, P.—(1923) *Virchows Arch.*, **244**, 1.

DIGBY, K. H., FOOK, W. L. AND CHE, Y. T.—(1941) *Brit. J. Surg.*, **28**, 517.
DJOJOPRANTO, M. AND MARCHETTA, F. C.—(1959) *Arch. Otolaryngol., Chicago*, **69**, 155.
DUNLAP, A. M.—(1938) *Chin. med. J.*, **53**, 68.
EDINGTON, G. E.—(1956) *Brit. J. Cancer*, **10**, 594.
ELMES, B. G. T. AND BALDWIN, R. B. T.—(1947) *Ann. trop. Med. Parasit.*, **41**, 321.
EWING, J.—(1929) *Amer. J. Path.*, **5**, 99.
FOY, H., KONDI, A., TIMMS, G. L., BRASS, W. AND BUSHRA, F.—(1954) *Brit. med. J.*, i, 204.
GELFAND, M.—(1949) *S. Afr. med. J.*, **23**, 1010.
GODTFREDSEN, E.—(1944) *Acta. path. microbiol. scand.*, Suppl. 50.
HOU, P. C.—(1960) *Rep. Brit. Emp. Cancer Campgn.*, **37**, 592.
HSIAO SHIH-CHIH, CHAO CHIA-FANG, WANG T'ING-FANG AND WANG YUNG-FU.—(1959) *Chin. med. J.*, **79**, 46.
HU CHENG-HSIANG AND YANG CHIEN.—(1959) *Ibid.*, **79**, 409.
LAMBERT, V.—(1960) *J. Laryng.*, **74**, 1.
MARSDEN, A. T. H.—(1958) *Brit. J. Cancer*, **12**, 161.
MCNEILL, K. A.—(1960) From ' Transactions of the 7th Pan-American Congress of Oto-Rhino-Laryngology and Bronchoesophagology'.
MARTIN, H. AND QUAN, S.—(1951) *Ann. Otol., etc., St. Louis*, **60**, 168.
MARTIN, H. E.—(1946) ' Cancer of the nasopharynx, in diseases of the nose, throat and ear.' Philadelphia & London (Saunders) p. 188.
MUIR, C. S.—(1962) *Brit. J. Cancer*, **16**, 583.
O'CONOR, G. T.—(1961) *Cancer*, **14**, 270.
ORMEROD, F. C.—(1951) *J. Laryng.*, **65**, 778.
OSBORN, D. A.—(1956) *Ibid.*, **70**, 574.
PANG, L.—(1959) *Ann. Otol., etc., St. Louis*, **68**, 356.
QUICK, D. AND CUTLER, M.—(1927) *Surg. Gynec. Obstet.*, **45**, 320.
REGAUD, C. AND REVERCHON, L.—(1921) *Rev. Laryng.*, Paris, **42**, 369.
RINGERTZ, N.—(1938) *Acta Otolaryng., Stockh.*, Suppl. 27.
SCHMINCKE, A.—(1921) *Beitr. path. Anat.*, **68**, 161.
SHAPIRO, M. P., KEEN, P., COHEN, L. AND MOOR, N. G.—(1955) *S. Afr. med. J.*, **29**, 95.
STEINER, P. E.—(1954) ' Cancer : Race and Geography '. Baltimore (Williams & Walkins) p. 307.
WILLIS, R. A.—(1960) ' Pathology of tumours.' London (Butterworth) p. 302.
YEH, S.—(1962) *Cancer*, **15**, 895.
ZIPPIN, C., TEKAWA, I. S., BRAGG, K. U., WATSON, D. A. AND LINDEN, G.—(1962) *J. nat. Cancer Inst.*, **29**, 3.